# Editorial

**Published:** 1890-11

**Authors:** 


### Editorial.

### THE SPECIALIST.

At a social gathering recently my attention was drawn to the Specialist himself. I sat for a long time listening to the conversation of three whose work is limited to nearly the same region.

A case was brought up, and each gave his opinion. I, myself, having some experience in two of the branches of medicine or surgery under discussion, I will not say which, was rather amused. Now, the question arises, what is a specialist? In the name of another I would to him asking the question, answer: "Show me the man who can treat a square inch of the body perfectly, and I will show you a man who can treat the whole body well." You may, perhaps, say I am drawing an ideal. Not so; for I believe the specialist is *made*, not *born*. By this I mean that every specialist should have a *thorough* medical education, as well as a good deal of practical medical and surgical experience before he launches out as a specialist, for otherwise how can he tell which branch he is best fitted for? The man who takes a six weeks' special course in surgery, genito-urinary disease, diseases of women, eye, ear, etc., is no more a specialist in that branch than a new-born babe.

Take the names of some of our greatest men; what do they show? That they were not masters of one branch alone, but that they were competent in all.

Take our own late lamented Westmoreland. Was he simply a surgeon? No; far from it. He was one of the best diagnosticians and therapeutists that the country has ever produced. It was the same with Sands, Wood, Agnew and many others.

The Specialist must, to be successful, have a full and thorough knowledge of medical science, or how can he tell which symptoms or diseases are reflex or otherwise? Take the different symptoms arising from a contracted meatus, a strictured rectum, a flexed uterus, or hypermetrophic nasal passage.

The general practitioner who could find time to treat, or afford the instrumental means to successfully combat all diseases, must of necessity be rare indeed

It will be seen from the above that the specialist is a necessity. He and the general practitioner should be the best of friends, for both need the aid of the other at times.

At times, it is true, the one must go beyond his field, as, for example, an eye specialist must give some attention to the general system, in case it becomes slightly deranged during his course of treatment, thus showing again the necessity of a thorough medical education.

The tendency of the day is toward specialties. It is, I fear, to some extent, over done, and the young man who enters the college with the idea of becoming a surgeon, or rectal specialist, should be very careful, for he is going to do that which, in nine cases in ten, he is not fitted for. Let him become a specialist in all branches of the medical science first, then he will not fail.

To the specialist I would say, go on in your grand career; be like the eagle and soar, and the soarer you get, the more we shall be gratified. F. O. S.

---

THE AMERICAN PUBLIC HEALTH ASSOCIATION will hold its eighteenth annual meeting in Charleston, South Carolina, on the 16th, 17th, 18th and 19th of December, under the presidency of Dr. Henry B. Baker, of Lansing, Michigan.

---

### SOUTHERN SURGICAL AND GYNECOLOGICAL ASSOCIATION.

The third annual meeting of the Southern Surgical and Gynecological Association will convene in Concordia Hall, Atlanta, Ga., on the 11th of November, and remain in session for three days.

In noticing the coming meeting, we take occasion to call attention to the excellent work that this body has accomplished at the two previous meetings, and to commend it to the profession of this section, as representing what we can accomplish in giving prominence to the high class of work that is emanating from the South. It lies with the profession to make the Association even a more conspicuous success than it has already shown itself.

As far as the work of the coming meeting is concerned, we would refer to the official programme to show that there will be little time for anything but work, yet the Committee of Arrangements has prepared a programme that will afford much opportunity for the cultivation of the pleasant social relations that count for so much in the maintaining of similar bodies everywhere.

Among the papers to be read are the following, as taken from the report of the Secretary:

PAPERS TO BE READ—FARTIAL LIST.

The President's Annual Address—George J. Englemann, M. D., St. Louis, Mo.

.How Shall We Treat Our Cases of Pelvic Inflammation?—R. B. Maury, M. D., Memphis, Tenn.

The General and Local Treatment of Gangrenous Diseases and Wounds—Bedford Brown, M. D., Alexandria, Va.

Further Study of the Direct and Reflex Effects of Lacerations of the Female Perineum—J. H. Blanks, M. D., Nashville, Tenn.

Abdominal and Pelvic Surgery in America—Joseph Price, M. D., Philadelphia, Pa.

Intra Ligamentous Ovarian Cystoma—Cornelius Kollock, M. D., Cheraw, S. C.

Anatomy and Pathology of the Ileo-Caecal Region—Richard Douglas, M. D., Nashville, Tenn.

Wet Antiseptic Dressings in Hand Injuries—Wm. Perrin Nicholson, Atlanta, Ga.

The Best Route to the Bladder in the Male for Disease or for Foreign Bodies—Hunter McGuire, M. D., Richmond, Va.

Suprapubic Cystotomy in a Case of Enlarged Prostrate—Wm. H. H. Cobb, M. D., Goldsboro, N. C.

Indications for Cholecystotomy—A. M. Owen, M. D., Evansville, Ind.

Uterine Moles and Their Treatment—J. T. Wilson, M. D., Sherman, Texas.

Strictures of the Male Urethra—W. F. Westmoreland, M. D., Atlanta, Ga.

Treatment of Urethral Strictures by Electricity—W. Frank Glenn, M. D., Nashville, Tenn.

The Surgical Treatment of Empyema—J. A. Goggans, M. D., Alexander City, Ala.

Cases in Abdominal Surgery—I. S. Stone, M. D., Lincoln, Va.

Rectal Medication in Pelvic Troubles—W. Hampton Caldwell, Lexington, Ky.

Conservarive Surgery in Injuries of the Foot—J. T. Wilson, M. D., Sherman, Texas.

The Management of the Infantile Prepuce—George Ben. Johnson, Richmond, Va.

The Ultimate Results of Trachelorrhaphy—Virgil O. Hardon, Atlanta, Ga.

Further Observations on the Dangers of Operative Delay in Prostatic Troubles, with Personal Experience—R. D. Webb, M. D., Birmingham, Ala.

Clinical History of the Epicystic Surgical Fistula—Jno. D. S. Davis, M. D., Birmingham, Ala.

Foreign Bodies in the Air Passages, with Report of Cases—John E. Pendleton, M. D., Hartford, Ky.

Chlecystomy—W. E. B Davis, M. D., Birmingham, Ala.

Two cases of Laporatomy for Intestinal Obstruction—J. T. Jelks, M. D., Hot Springs, Ark.

Is Gonorrhoea Ever a Cause for Pelvic Inflammations?—J. G. Griggs, M. D., Birmingham, Ala.

Treatment of General Septic Peritonitis—W. L. Robinson, M. D., Danville, Va.

A Case of Fractnre of the Femur, due to Fragility—Hunter P. Cooper, M. D., Atlanta, Ga.

Inflammation in and about the Head of the Colon—L. S. McMurtry, M. D., Louisville, Ky.

Removal of Stone from Female Bladder through the Urethra, with Cases—W. O. Roberts, M. D., Louisville, Ky.

A New Jacket for the Treatment of Spinal Diseases and Injuries—G. A. Baxter, M. D., Chattanooga, Tenn.

A Review of the Treatment of Varicocele, with Cases—G. Frank Lydston, M. D., Chicago, Ill.

Exhibition of Pathological Specimens Removed by Laparotomy—W. H. Wathen, M. D., Louisville, Ky.

A Case of Strangulated Umbilical Hernia complicated with a large Uterine Fibroid, Supravaginal Hysterectomy and Operation for Hernia—Joseph Taber Johnston, M. D., Washington, D. C.

The Indications for Operation in Ectopic Gestation—Chas. A. L Reed, M. D., Cincinnati, Ohio.

The Indiscriminate use of Opium in the Pelvic Disease of Women—H. P. C. Wilson, M. D., Baltimore, Md.

Report of Two Cases of Cranial Surgery—Henry L. Fountain, M. D., Bryain, Texas.

Some Observations on Rectal Surgery—Shep. A. Rogers, M. D., Memphis, Tenn.

Caneer of the Cervix Uteri in the Negress—Howard Kelly, M. D.‘ Baltimore, Md.

A glance over the titles shows the interesting material that will be brought before the body, and we predict that the meeting will be equal in interest and value to any in this country. Any informstion in reference to the Association can be obtained by addressing Dr. W. E. B. Davis, Birmingham, Ala. The members of the profession are cordially invited to be present.

---

### THE SANITARIAN.

A Monthly Magazine, devoted to the Preservation of Health, Mental and Physical Culture. $4.00 a year. 35 cents a number. Sample copies 20 cents—10 two-cent postage stamps. A. N. BELL, A. M., M. D., Editor, 113A Second Place, Brocklyn, New York.

The American News Company, Nos. 39 and 41 Chambers St., New York, General Agents. Newsdealers will send orders to them.

Correspondents—*All* communications, except from Newsdealers, all exchanges with the *Sanitarian* and all books for review, should be addressed to the Editor. Remittances should be made either by postoffice order, registered letter, bank check or draft on New York or Brooklyn, to the order of A. N. Bell.

